# Identification of Antiglycative Compounds in Japanese Red Water Pepper (Red Leaf Variant of the *Persicaria hydropiper* Sprout)

**DOI:** 10.3390/molecules23092319

**Published:** 2018-09-11

**Authors:** Wakako Takabe, Taiki Yamaguchi, Hideharu Hayashi, Natsuhiko Sugimura, Masayuki Yagi, Yoshikazu Yonei

**Affiliations:** 1 Antiaging Medical Research Center and Glycation Stress Research Center, Graduate School of Life and Medical Sciences, Doshisha University, 1-3 tatara Miyakodani, ktotanabe City, Kyoto 610-0394, Japan; ctub2025@mail4.doshisha.ac.jp (T.Y.); ctub2004@mail4.doshisha.ac.jp (H.H.); myagi@mail.doshisha.ac.jp (M.Y.); yyonei@mail.doshisha.ac.jp (Y.Y.); 2 Materials Characterization Central Laboratory, Waseda University, 3-4-1 Ohkubo, Shinjyuku, Tokyo 169-8555, Japan; sugimura@waseda.jp

**Keywords:** glycation, *Persicaria hydropiper*, *Polygonum hydropiper*, Benitade, formation of AGEs, idaein, hyperin

## Abstract

Glycation, the nonenzymatic reaction between proteins and excess blood sugar, is implicated in multiple disorders and occurs via the formation and accumulation of advanced glycation end products (AGEs). In our previous studies, we demonstrated that the red-leaf variant of the *Persicaria hydropiper* sprout (Japanese red water pepper, Benitade) is one of the potent plants that inhibit formation of AGEs. In this study, we aimed to identify antiglycative compounds in Benitade. Benitade extracts were prepared with hot water, then fractionated by using high-performance liquid chromatography (HPLC). The antiglycative efficacy of each fraction was evaluated by measuring the formation of fluorescent AGEs (Ex 370 nm/Em 440 nm). Two fractions, which contained peaks at 26.4 min and 31.8 min, showed potent antiglycative efficacy. When we hydrolyzed these peaks, they shifted to 32.5 and 41.4 min, which are the same retention times as cyanidin and quercetin, respectively. Based on thin-layer chromatography, both compounds contained galactose. Finally, ultrahigh-performance liquid chromatography/quadrupole-time of flight mass spectrometry (UHPLC-QqTOF-MS) analyses were performed to determine the structure of those compounds. Overall, we identified two glycosides, cyanidin 3-*O*-galactoside (idaein) and quercetin 3-*O*-galactoside (hyperin), as representative antiglycative compounds in Benitade.

## 1. Introduction

In mammals, high blood glucose is the leading cause of glycation, the nonenzymatic reaction between proteins and a reducing agent, and it forms advanced glycation end products (AGEs). Even in a normal metabolism, AGEs are produced and accumulated spontaneously in human tissue [1]. Food intake may also cause accumulation of AGEs in human body [2]. Usually, AGEs are removed through enzymatic clearance and renal excretion; however, with aging, there is an imbalance between the formation and clearance of AGEs, leading to their accumulation [3]. Several lines of evidence have shown that accumulation of AGEs mediate age-related diseases, such as cancer [4], diabetes mellitus [5], osteoporosis [6], Alzheimer’s disease [7,8], and cardiovascular disease [9]. From that point of view, the inhibition or decomposition of AGEs has been studied to relieve disease symptoms. Although several chemical inhibitors have been established, none has been approved in Japan due to serious side effects. Therefore, natural antiglycative compounds from vegetables and fruit have been identified in these decades. 

Even when same-term AGEs were used, many kinds of AGEs exist because of the difference in proteins and reducing agents. To evaluate the effect of plants/compounds against glycationthe fluorescence, excitation of 370 nm, and emission at 440 nm are widely used for measuring the amount of AGEs. That method cannot actually detect fluorescence, but different excitation wavelengths are required for AGE such as pentosidine (excitation at 335 nm, emission at 385 nm) and nonfluorescent AGE *N^ε^*-(carboxymethyl)lysine (CML). Pentosidine was elevated in osteoporosis patients [10] and CML levels were increased in diabetes patients [11]. The measurement of specific AGE is valuable in discussing the contribution of specific AGEs on particular diseases, but these are complicated and costly methods. Instead of that, Meerwaldt et al. reported that the levels of skin autofluorescence (excitation at 300–420 nm, emission at 420–600 nm) were related to AGE levels in dermal biopsies in diabetes patients and healthy controls [12]. Skin autofluorescence levels were also increased in renal disease [13]; that means the measurement of skin fluorescence may be useful as a biomarker in AGE-related diseases [14]. Fluorescent AGEs (Ex 370/Em 440 nm) were increased in the blood, urine [15], and dura mater [16] of diabetic patients. Based on these clinical data, many research groups have used the amount of fluorescent AGEs (Ex 370/Em 440 nm) to examine the efficacy of inhibiting glycation reaction [17,18,19].

We have used over 500 kinds of plants to examine their efficacies against the formation of fluorescent AGEs (antiglycative effect) using in vitro glycation models, by which glucose is tested with one of the most abundant blood proteins: human serum albumin (HSA) [20,21,22,23,24]. Furthermore, we investigated the antiglycative effect of 73 kinds of plants with not only the HSA glycation model but also type I collagen and type II collagen glycation models [25], and we demonstrated that the red leaf variant of the *Persicaria hydropiper* (Syn. *Polygonum hydropiper*) sprout (Japanese red water pepper, Benitade) is a potent antiglycative plant against the formation of fluorescent AGEs and glycation intermediates in those three glycation models.

*P. hydropiper* belongs to the Polygonaceae family and is used in folk medicine against cancer [26]. One of the principal compounds, sesquiterpene dialdehyde (polygodial), has a spicy taste [27], as well as antibacterial efficacy [28,29]. Due to its antibacterial effect, Benitade is generally used as spice with raw fish in Japan. Multiple flavonoids have also been identified in *P. hydropiper*, including quercetin, 6-hydroxyluteorin, and their glycosides [30]; isoquercitrin, specifically, is reported to be a potent antioxidative compound [31]. However, the antiglycative effects of Benitade have not been well-studied.

In this study, we aimed at identifying the effective antiglycative compounds in Benitade.

## 2. Results and Discussion

### 2.1. Benitade Extracts Inhibited Formation of Fluorescent AGEs in a Dose-Dependent Manner

**Figure 1:**
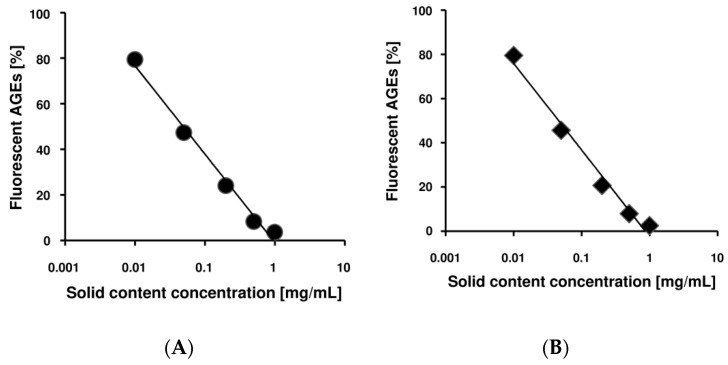
Effect of *Persicaria hydropiper* extracts on formation of fluorescent advanced glycation end products (AGEs). *P. hydropiper* extract of either (**A**) water or (**B**) 70% (*v*/*v*) ethanol (EtOH) was introduced into the human serum albumin (HSA) glycation models at the indicated solid-content concentrations. After 40 h incubation at 60 °C, fluorescent AGEs were measured at 370/440 nm.

First, to investigate the solubility of the Benitade compounds effective against glycation, we used two different kinds of solvent, i.e., water and 70% (*v*/*v*) ethanol (EtOH). Both extracts were prepared from 2 g of dried Benitade powder and 40 mL solvent, from which we collected 35 mL of solution. By water extraction, 17.5 mg/mL solid content was obtained, and for 70% (*v*/*v*) EtOH, 14.8 mg/mL solid content was acquired. Then, we adjusted those extracts to the indicated concentrations by either distilled water or 70% (*v*/*v*) EtOH and evaluated the antiglycative effects in the HSA glycation model. We incubated HSA and glucose with or without Benitade extracts at 60 °C for 40 h to evaluate their antiglycative effect. The glycation reaction is nonenzymatic, and is controlled by reaction temperature and time. In our previous reports, we demonstrated that the formation of fluorescent AGEs at 60 °C for 40 h was equivalent to that obtained from a reaction time of about 60 days at 37 °C [32]. As shown in Figure 1, Benitade extract inhibited formation of fluorescent AGEs in a dose-dependent manner. The water extract (A) and 70% (*v*/*v*) EtOH extract (B) showed no difference in inhibitory efficacy (IC_50 water_ = 49 μg/mL, IC_50 70% EtOH_ = 46 μg/mL). These data suggest that the major effective antiglycative compound in Benitade might be hydrophilic. Therefore, we used the water extract of Benitade (we named it PEW (*P. hydropiper* extracts by water) in this manuscript) for further studies to determine the antiglycative compounds.

### 2.2. Isolation of Antiglycative Compounds in Benitade Extract

**Figure 2:**
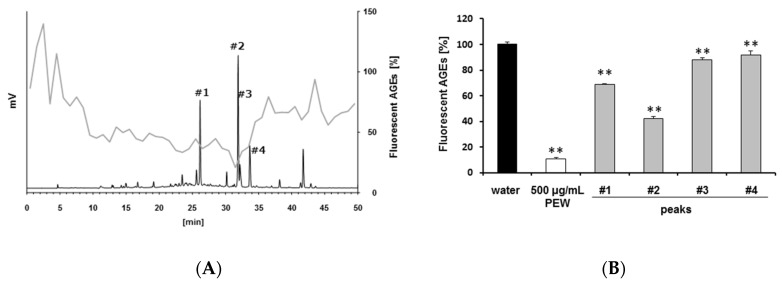
Isolation of antiglycative compounds in *Persicaria hydropiper*. PEW was fractionated using high-performance liquid chromatography ultraviolet (HPLC-UV). Fractions were collected every minute. (**A**) Black line: HPLC chromatogram at 270 nm. Gray line: the efficacy of fractionated samples against formation of fluorescent AGEs. (**B**) Two-hundred microliters of 5 mg/mL solid-content concentration of PEW was used to isolate these four peaks and was concentrated to around 50 μL by using a rotary evaporator. Then, each solution was adjusted to 200 μL by water. To compare the effect of individual peaks with the whole PEW efficacy, 5 mg/mL solid content of PEW was also used. The whole PEW and the indicated isolated peaks were added as 10% volume content to the HSA glycation model. After 40 h incubation at 60 °C, fluorescent AGEs were measured at 370/440 nm. ** *p <* 0.01 vs. water.

To isolate the compounds in PEW, 200 μL PEW (5 mg/mL solid content concentration) was separated by HPLC, and the flow-through was collected every minute. Each fractionated flow-through was concentrated by evaporation and adjusted to 200 μL by using distilled water. Then, every fraction was evaluated for its antiglycative efficacy. Formation of fluorescent AGEs was strongly inhibited by several fractions, such as Fr. 27 (retention time (RT) = 26.00–26.99 min) and Fr. 32 (RT = 31.00–31.99 min) (Figure 2A, gray line). When we analyzed the fractions at UV 270 nm, one of the typical absorbances of polyphenols, several peaks were observed, including 26.4, 31.8, 32.2, and 33.8 min (Figure 2A, black line peaks #1–4).

Next, we also used 200 μL PEW (5 mg/mL solid content concentration) to isolate peaks 1–4 by hand. After concentration and adjustment to 200 μL by distilled water, investigations of the antiglycative efficacy of every peak were conducted. As shown in Figure 2B, all four peaks significantly inhibited formation of fluorescent AGEs. Among them, peaks 1 and 2 were much more effective than the other peaks, even though every peak was less effective than the whole PEW. These results suggest that multiple antiglycative compounds were contained in the PEW.

### 2.3. Identification of Antiglycative Compounds in Benitade Extract

**Figure 3:**
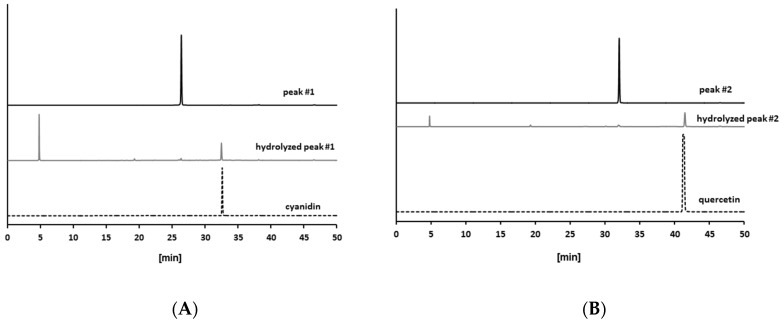

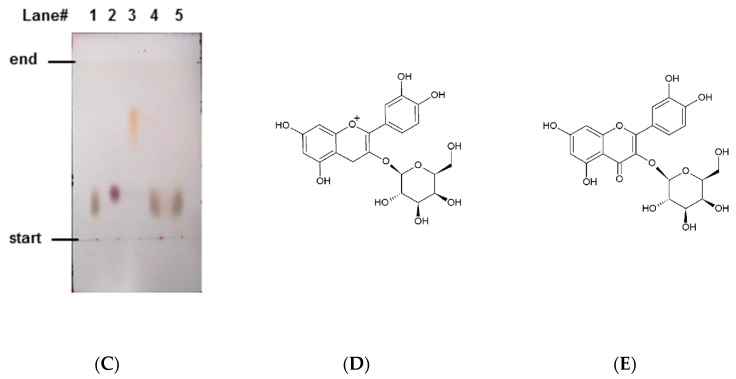
Identification of glycosides as antiglycative components of *Persicaria hydropiper.* (**A**,**B**) HPLC-UV chromatogram at 270 nm. (**A**) Isolated peak 1: before hydrolysis (black line), after hydrolysis (gray line), authentic standard of cyanidin (4 nmol, broken line). (**B**) Isolated peak 2: before hydrolysis (black line), after hydrolysis (gray line), authentic standard of quercetin (1 nmol, broken line). (**C**) Thin-layer chromatogram for saccharides. Lane 1, galactose (10 nmol); lane 2, glucose (10 nmol); lane 3, rhamnose (10 nmol); lane 4, hydrolyzed peak 1; lane 5, hydrolyzed peak 2. (**D**,**E**) Chemical structures of (**D**) cyanidin-3-*O*-galactoside (idaein) and (**E**) quercetin-3-*O*-galactoside (hyperin).

Because multiple antiglycative compounds in PEW had an absorption at 270 nm (Figure 2), we speculated that those compounds contain polyphenols. Other than natural polyphenols in vegetables and fruit, glycosides, in which various sugar moieties are bound to polyphenols via a glycosidic bond, are known to be potent antioxidants [30,31,33]. Oxidation is one of the critical steps in glycation, and several antioxidative polyphenols and glycosides, such as procyanidin [34], luteolin, and quercetin 3-*O*-rutinoside (rutin) [35], have been reported as antiglycative compounds. Several glycosides have also been reported for their protective effects in diabetic complications. Iridoid glycoside (morroniside) extracted from *Corni fructus* ameliorated diabetic hepatic complications in db/db mice [36]. Guimaraes et al. reported that rutin attenuated cardiac structural changes and myocardial dysfunction in rats with type I diabetes [37]. Thus, we investigated whether or not antiglycative compounds in the PEW were glycosides. We hydrolyzed peaks 1 and 2 by using hydrochloric acid (HCl) with heating, and compared their HPLC chromatograms with those of typical polyphenols in plants. As shown in Figure 3A, peak 1 moved from 26.5 to 32.5 min after hydrolyzation (black line to gray line). We also analyzed major polyphenols and authentic standard of cyanidin showed its RT as 32.5 min (broken line). Similar to peak 1, peak 2 shifted from 31.8 min to 41.4 min, the same RT as authentic standard of quercetin (Figure 3B). These data indicate that both peaks contain polyphenols, either cyanidin or quercetin, as aglycones.

Next, we investigated the saccharides which were incorporated into the extracted glycosides. Hydrolyzed samples were spotted and isolated by using thin-layer chromatography (TLC). As shown in Figure 3C, both hydrolyzed peaks had the same retention factor (Rf = 0.17) and color (grayish brown) as galactose, while they differed from glucose (Rf = 0.21, violet) and rhamnose (Rf = 0.66, orange). These data indicate that the compound in peak 1 might be assigned as cyanidin 3-*O*-galactoside (idaein, chemical structure in Figure 3D), and the compound in peak 2 was tentatively identified as quercetin 3-*O*-galactoside (hyperin or hyperoside, Figure 3E).

**Figure 4:**
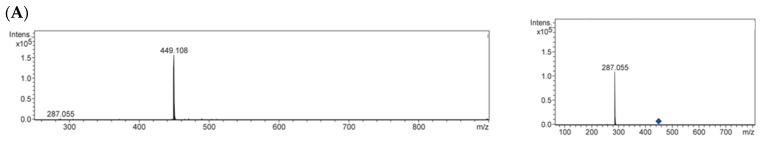

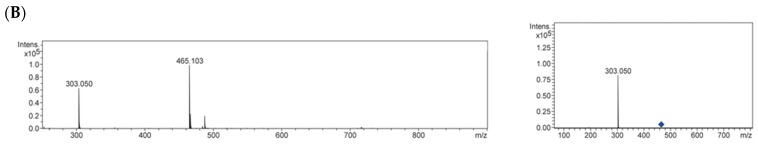
UHPLC-QqTOF-MS analyses of antiglycative components in *Persicaria hydropiper.* (**A**) Mass spectrum (left panel) and MS/MS spectrum (right panel) of peak 1. (**B**) Mass spectrum (left panel) and MS/MS spectrum (right panel) of peak 2.

We further performed ultrahigh-performance liquid chromatography/quadrupole-time of flight mass spectrometry (UHPLC-QqTOF-MS) analyses to elucidate the structure of these compounds. In positive mode, peak 1 produced a pseudomolecular ion at *m/z* 449.108 (Figure 4A, left panel), the equivalent *m/z* to idaein. MS/MS analysis of the peak gave an aglycone (cyanidin) cation at *m/z* 287.055 (Figure 4A, right panel). Peak #2 gave a protonated molecule [M + H]^+^ of hyperin at *m/z* 465.103 (Figure 4B, left panel) and the peak produced an [M + H]^+^ fragment ion at *m/z* 303.050 (Figure 4B, right panel), the identical *m/z* to quercetin. Overall, these data suggest that, in PEW, idaein and hyperin are major compounds to inhibit formation of fluorescent AGEs. Several studies in the literature have demonstrated that idaein is one of the major flavonoids in Benitade [38], and hyperin is contained in *Fagopyrum esculentum* [39], which belongs to the Polygonaceae family, to which Benitade also belongs.

**Figure 5:**
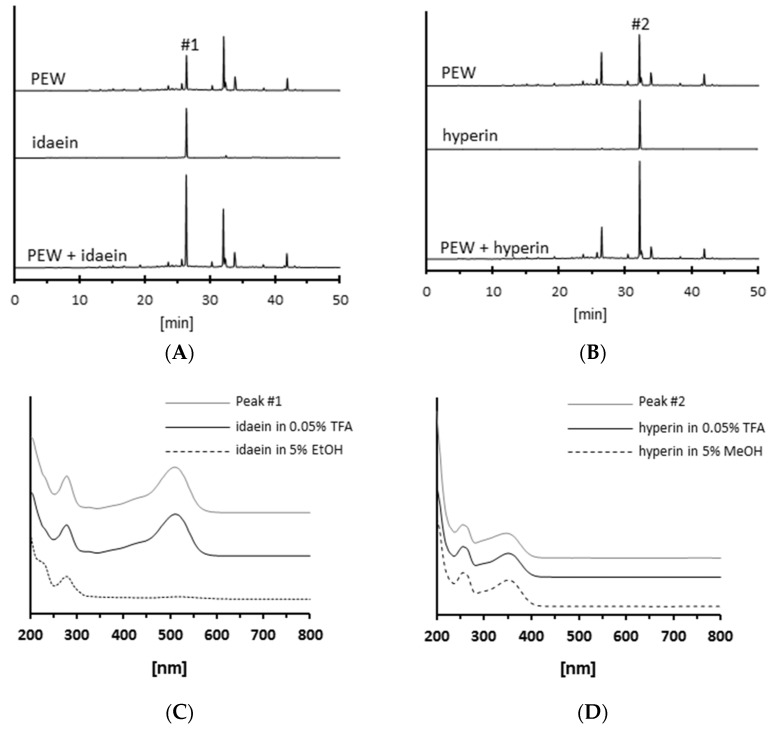
Comparison of the isolated peaks from *Persicaria hydropiper* extract with idaein and hyperin. (**A**,**B**) Ten micrograms of PEW was mixed with either authentic standard of idaein or hyperin (0.4 nmol) and analyzed using HPLC-UV at 270 nm. (**C**,**D**) UV-vis absorption spectrum of the isolated peaks and authentic standards of idaein and hyperin.

In the next series of experiments, we compared the isolated peaks from the PEW with authentic standards of idaein and hyperin. In HPLC-UV analyses, idaein and hyperin showed RTs of 26.4 and 31.8 min, the same RTs of peaks 1 and 2 (Figure 5A,B). When we mixed these glycosides (0.4 nmol each) with 10 μg PEW, peaks 1 and 2 increased. Furthermore, we compared the UV-vis absorption spectrums (Figure 5C,D). Isolated peak 1 had maximum absorption bands at around 270 and 510 nm (Figure 5C, gray line), while authentic standard of idaein absorbed at 270 nm but not 510 nm in 5% (*v*/*v*) EtOH, which was at neutral pH (Figure 5C, broken line). However, the authentic standard of idaein showed absorption at around 270 and 510 nm when it was in an acidic condition like the eluent, which was used for HPLC-UV analyses (0.05% (*v*/*v*) trifluoroacetic acid (TFA), Figure 5C, black line). Isolated peak 2 and authentic standard of hyperin showed 250 and 350 nm maximum absorption, regardless of pH condition (Figure 5D). Overall, these data suggest that isolated peaks 1 and 2 from the PEW were idaein and hyperin, respectively.

### 2.4. Idaein and Hyperin and Their Aglycones Inhibited Formation of Fluorescent AGEs in a Dose-Dependent Manner

By using HPLC-UV, we determined the concentration of idaein and hyperin in the PEW. Consequently, 72 nmol (34.5 μg) idaein and 35 nmol (16.4 μg) hyperin were contained in 1 mg solid content of PEW. That means that 49 μg/mL solid content of PEW—the IC_50_ against formation of fluorescent AGEs (Figure 1A)—contained 3.5 μmol/L idaein and 1.6 μmol/L hyperin.

**Figure 6:**
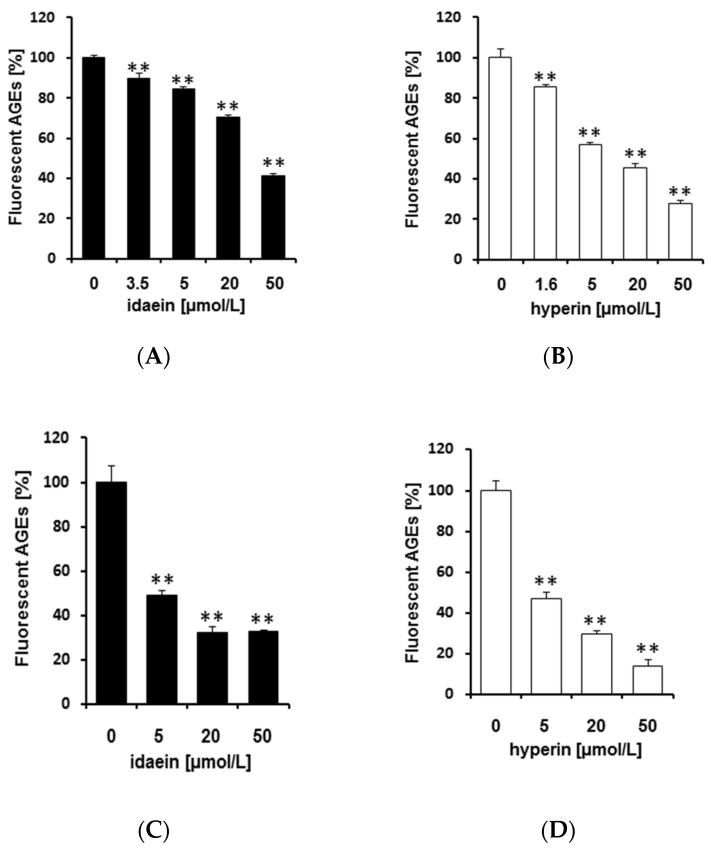
Efficacy of idaein and hyperin against formation of fluorescent AGEs. The indicated concentrations of authentic standards of idaein and hyperin were used to determine the inhibitory effect against formation of fluorescent AGEs in the (**A**,**B**) HSA glycation model and (**C**,**D**) type I collagen glycation model. After 40 h incubation at 60 °C, fluorescent AGEs were measured at 370/440 nm. ** *p <* 0.01 vs. 0 μmol/L.

In this experiment, we evaluated the efficacy of authentic standards of idaein and hyperin against formation of fluorescent AGEs. As shown in Figure 6A,B, both idaein and hyperin inhibited formation of fluorescent AGEs in the HSA glycation model in a dose-dependent manner. We also evaluated the efficacy of 3.5 μmol/L idaein and 1.6 μmol/L hyperin, which are contained in the IC50 of PEW, and the efficacies were around 10% and 15% inhibition, respectively. Previously, we demonstrated that Benitade is a potent antiglycative plant not only in the HSA glycation model but also in the collagen glycation model [25]. Thus, we also investigated the effect of these glycosides against glycation of type I collagen. Both idaein and hyperin suppressed formation of fluorescent AGEs in the type I collagen glycation model, as well as in HSA glycation models (Figure 6C,D). These data indicate that idaein and hyperin substantially contribute to PEW’s antiglycative efficacy.

We also calculated the yield of idaein and hyperin from fresh Benitade. When we dried 100 g of fresh Benitade, 12.8 g of dried Benitade was obtained. That means, based on the calculation of solid content of PEW, if we prepare PEW from 100 g of fresh Benitade, 3920 mg of solid content should be acquired. In 1 mg of solid content of PEW, 34.5 μg idaein and 16.4 μg hyperin were contained, so we may obtain 135.2 mg of idaein and 64.3 mg of hyperin. The yield of these compounds in PEW from fresh Benitade was 0.14% (*w*/*w*) and 0.06% (*w*/*w*), respectively.

In this study, we investigated the efficacy of glycosides in water extract of *Persicaria hydropiper* against formation of fluorescent AGEs. However, for intestinal absorption, glycosides are first hydrolyzed by glycosidases in the small intestine, then absorbed as aglycones. Thus, we further examined the aglycones of idaein and hyperin: cyanidin and quercetin. When we compared these glycosides and aglycones, hyperin itself and quercetin—an aglycone of hyperin—had more potent antiglycative effects than idaein and cyanidin—an aglycone of idaein (Figure 6B and Figure S1B vs. Figure 6A and Figure S1A). These finding indicate that regardless of whether it has a glycone (saccharide), quercetin structures are stronger than cyanidin structures against glycation. Oxidation plays a critical role in the early steps of glycation; thus, we firstly speculated that the antioxidative efficacy may contribute to their antiglycative effect. However, Kähkönen et al. demonstrated that the radical scavenging activity of aglycones against the 2,2-diphenyl-1-picrylhydrazyl (DPPH) radical is not significantly different between cyanidin and quercetin [33]. Ioku et al. reported that quercetin exhibited more potent peroxyl radical-scavenging activity than the glycoside [40]. Wang et al. also performed the oxygen radical absorbance capacity (ORAC) assay to evaluate the scavenging activities of peroxyl radicals, and they showed cyandin had more potent antioxidant activity than idaein [41]. Interestingly, they also tested other glycosides, such as cyanidin-3-glucoside (kuromanin) and cyanidin-3-rhamnoglucoside (keracyanin), which showed strong antioxidant activity that corresponding aglycone, cyanidin. These data indicates that depend on the sugars may have different effects on the antioxidant activity.

Based on our data, idaein and hyperin tend to show a more potent antiglycative effect than their corresponding aglycones, especially in lower concentrations (Figure 6A vs. Figure S1A, Figure 6B vs. Figure S1B). This information indicates that the antiglycative efficacy is not solely due to its antioxidative effect. Further study is needed to clarify the relationship between antiglycative effects and antioxidative effects. 

Idaein, which is a combination of galactose and cyanidin, contributes to the red color of Benitade. Benitade, which is a variant of *P. hydropiper* (red leaf), and the original green sprout of *P. hydropiper* (Aotade in Japanese), does not contain idaein. Therefore, Benitade may acquire the cyanidin synthesis pathway during cultivation, but further study is needed to clarify the difference between them. Haraguchi et al. reported that quercetin 3-*O*-glucoside (isoquercitrin) is a major antioxidative compound in Benitade [31]. In our HPLC conditions, the RT of isoquercitrin was 32.2 min (Figure S2, bottom chromatogram), the same RT as antiglycative peak 3 (Figure 2A). When 0.4 nmol isoquercitrin standard was mixed with 10 μg PEW, only peak 3 was increased (Figure S2, top chromatogram). Even though we have not had further confirmation, if peak 3 were isoquercitrin, 18.0 nmol (8.3 μg) was contained in 1 mg solid content of PEW based on HPLC-UV analyses. That means that, in 49 μg/mL solid content of PEW, the IC_50_ against formation of fluorescent AGEs (Figure 1A), 0.88 μmol/L was contained. Even though isoquercitrin inhibited formation of fluorescent AGEs at higher concentrations, such as over 10 μmol/L, 0.88 μmol/L did not show any inhibition (Figure S3). Furthermore, as shown in Figure 2B, the efficacy of peak 3 against formation of fluorescent AGEs was much less than peak 1 (idaein) and 2 (hyperin). These data indicate that isoquercitrin in Benitade might not be a major antiglycative compound.

Taken together, our data suggest that idaein and hyperin are representative compounds in Benitade that inhibit glycation.

## 3. Materials and Methods

### 3.1. Materials

Benitade was purchased from a local grocery market in Kyoto, Japan in December 2016. HSA, idaein, hyperin, and isoquercitrin were purchased from Sigma-Aldrich (St. Louis, MO, USA). Type I collagen was obtained from Nippi Inc. (Tokyo, Japan). All other chemicals were obtained from Wako (Osaka, Japan) as analytical grade.

### 3.2. Preparation of Persicaria Hydropiper Extract

Benitade was dried and ground, and then its extracts were prepared using either water or 70% EtOH (EtOH/distilled water = 70/30 (*v*/*v*)). Briefly, 2 g of dried powder was mixed with 40 mL distilled water or 70% (*v*/*v*) EtOH. For water extraction, the sample was incubated at 80 °C for 75 min. For 70% (*v*/*v*) EtOH extraction, the sample was incubated at 49 °C for 4 h. Extracted samples were centrifuged at 3350× *g* for 10 min and filtered using filter paper. Five milliliters of the extracts was used for measurement of solid content by evaporation at 120 °C for 2 h, and then the extracts were adjusted to their appropriate concentrations by either water or 70% (*v*/*v*) EtOH.

### 3.3. Measurement of Antiglycative Effect of Benitade Eextract

#### 3.3.1. Preparation of Glycated Proteins

An HSA glycation model was used to evaluate the effect of *P. hydropiper* on glycation. HSA (8 mg/mL) and 0.2 mol/L glucose in 50 mmol/L phosphate buffer (PB, pH 7.4) were incubated at 60 °C for 40 h (named “solution A”). Simultaneously, heated proteins without glucose were also prepared (solution B). To determine the effects of Benitade extract, the indicated concentrations of samples were introduced into the reaction mixture with or without glucose (solutions C and D, respectively). To determine the efficacy of the compounds against glycation in collagen protein, 0.6 mg/mL type I collagen and 0.4 mol/L fructose in 50 mmol/L PB (pH 7.4) were incubated at 60 °C for 24 h.

#### 3.3.2. Measurement of AGE-Derived Fluorescence

AGE-derived fluorescence was measured as reported previously [21]. Briefly, 200 μL of the reaction mixture was used to measure fluorescence at an excitation wavelength of 370 nm and an emission wavelength of 440 nm by a Varioscan^®^ Flash (Thermo Scientific, Waltham, MA, USA) microplate reader. The value was calculated using the equation below.

Ratios of AGEs-derived fluorescence (%) = {fluorescence of (solution C − solution D)/fluorescence of (solution A − solution B)} × 100

### 3.4. Isolation of Compounds in Benitade Extract

#### 3.4.1. HPLC-UV System for Analyses

A Shimadzu HPLC-UV system (Shimadzu Corporation, Kyoto, Japan) was used. The HPLC conditions were as follows: Column, YMC Pack ODS-AM, 250 mm × 6 mm I.D. column (YMC CO., LTD, Kyoto, Japan); eluent, a linear gradient of acetonitrile (0–50%B) in 0.1% (*v*/*v*) TFA for 50 min. Column temperature was 40 °C and the flow rate and detection wavelength were 1.0 mL/min and 270 nm, respectively. For fractionation, we injected 200 μL, and for the other purpose, we injected 20 μL.

#### 3.4.2. Fractionation of Benitade Extract by HPLC

A fraction collector (FRC-10A, Shimadzu) was connected to the HPLC-UV system to isolate the compounds in the PEW. The flow-through was collected every minute. Collected samples were concentrated by evaporation using a rotary evaporator (TOMY Digital Biology, Tokyo, Japan), and then used for further analyses.

### 3.5. Hydrolysis of Glycosides

Samples were mixed with the same volume of 2 mol/L HCl and heated at 90 °C for 60 min, then evaporated with rotary evaporator. Pellets were dissolved using distilled water and used for further analyses.

### 3.6. Thin-Layer Chromatography for Saccharides

Silica gel 70 F_254_ TLC Plate (5 × 10 cm, Wako, Osaka, Japan) was used for the detection of saccharides. The mobile phase was acetone/distilled water = 90:10. After separation for 10 min, the plate was dried and soaked in 0.5% (*w*/*v*) 1-naphtol in 50% methanol solution (methanol/distilled water = 50/50 (*v*/*v*)). Then, the plate was sprayed with concentrated sulfuric acid and heated until spots appeared. 

### 3.7. Measurement of UV-Vis Absorption Spectrum

Two-hundred microliter samples were used to measure the absorption spectrum between 200 nm and 800 nm by a Varioscan^®^ Flash microplate reader.

### 3.8. UHPLC-QqTOF-MS Analysis

Isolated peaks from the PEW by HPLC-UV were evaporated and redissolved by distilled water, and UHPLC-QqTOF-MS analyses were performed for identification of the structure of the compounds. We used the technical services of MASIS Inc. (Aomori, Japan). The analytical conditions are listed below.

UHPLC: UHPLC Full System Nexera (Shimadzu, Kyoto, Japan), TOF-MS: TOF-MS maXis 4G (Bruker, Billerica, MA, USA); Column: Grand C18-3WK (2.0 mmφ × 100 mm; MASIS, Aomori, Japan). The mobile phase consisted of (**A**) 0.1% (*v*/*v*) formic acid and (**B**) 5 mmol/L formic acid in methanol. The elution gradient was set as follows: 10% **B** (0 min), 60% **B** (20 min), 95% **B** (20.1 min), 95% **B** (27 min), 10% **B** (27.1 min); flow rate: 0.2 mL/min; ionization method: ESI; capillary voltage: 4500 V; nebulizer gas: nitrogen 1.8 bar; drying gas flow rate: nitrogen 7.5 L/min; drying gas temperature: 180 °C; measurement mass range: *m/z* 250~900.

### 3.9. Statistics

Data are expressed as mean ± S.D. of at least 3 independent experiments. The statistical analyses, performed by analysis of variance (ANOVA), were subjected to Dunnett’s test for multiple comparisons between each of the samples and control groups. Differences were considered significant at *p* values less than 0.05.

## Supplementary Materials

The following are available online, Figure S1: Efficacy of aglycons against formation of fluorescent AGEs, Figure S2: Comparison of isolated peaks from Persicaria hydropiper extract with isoquercitrin, Figure S3. Efficacy of isoquercitrin against formation of fluorescent AGEs.
