# Supplementary material for: Identification of Antiglycative Compounds in Japanese Red Water Pepper (Red Leaf Variant of the Persicaria hydropiper Sprout)
Source: Molecules. 2018 Sep 11;23(9):2319. doi: 10.3390/molecules23092319 (PMC6225476; doi:10.3390/molecules23092319)
Supplement: Supplementary file 1 [file molecules-23-02319-s001.pdf]

Supplementary Materials:

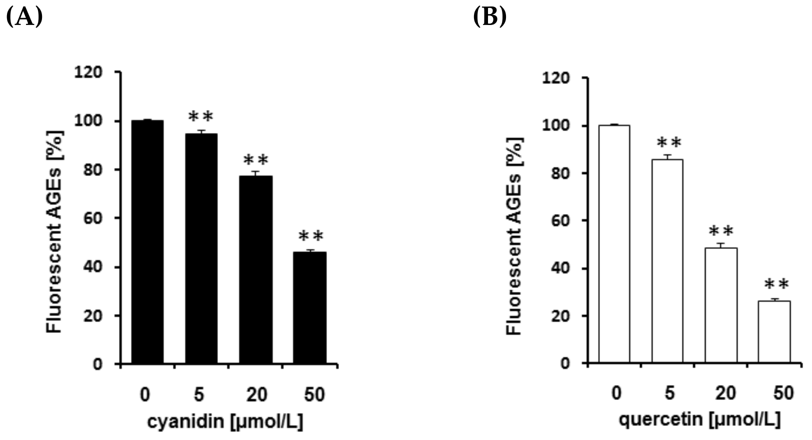

Figure S1. Efficacy of aglycons against formation of fluorescent AGEs. The indicated concentration of (A) cyanidin and (B) quercetin were used to determine their inhibitory effect against formation of fluorescent AGEs in the HSA glycation model. After 40 h incubation at 60 °C, fluorescent AGEs were measured at 370/440 nm. ** $p < 0.01$ vs. 0 µmol/L.

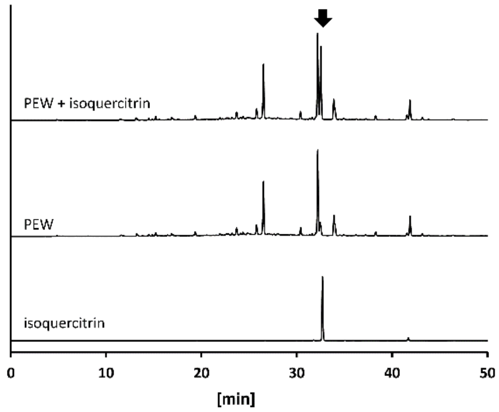

Figure S2. Comparison of isolated peaks from *Persicaria hydropiper* extract with isoquercitrin. Ten micrograms of PEW was mixed with isoquercitrin (0.4 nmol) and analyzed using HPLC–UV at 270 nm.

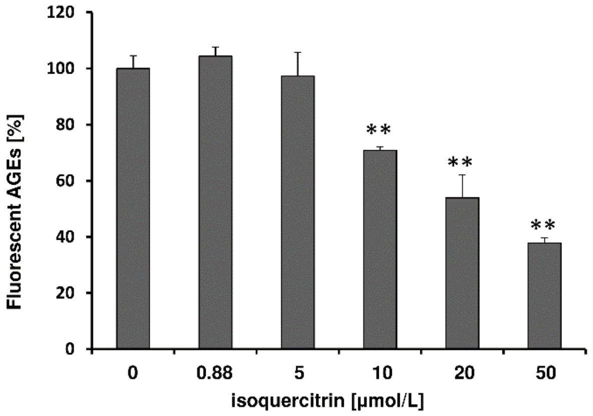

Figure S3. Efficacy of isoquercitrin against formation of fluorescent AGEs. The indicated concentration of isoquercitrin were used to determine the inhibitory effect against formation of fluorescent AGEs in the HSA glycation model. After 40 h incubation at 60 °C, fluorescent AGEs were measured at 370/440 nm. ** $p < 0.01$ vs. 0 µmol/L.
